# Impact of PI3K/AKT/mTOR pathway activation on the prognosis of patients with head and neck squamous cell carcinomas

**DOI:** 10.18632/oncotarget.8957

**Published:** 2016-04-23

**Authors:** Darío García-Carracedo, Maria Ángeles Villaronga, Saúl Álvarez-Teijeiro, Francisco Hermida-Prado, Iñigo Santamaría, Eva Allonca, Laura Suárez-Fernández, Maria Victoria Gonzalez, Milagros Balbín, Aurora Astudillo, Pablo Martínez-Camblor, Gloria H. Su, Juan Pablo Rodrigo, Juana María García-Pedrero

**Affiliations:** ^1^ Herbert Irving Comprehensive Cancer Center, Columbia University Medical Center, New York, NY, USA; ^2^ Department of Otolaryngology, Hospital Universitario Central de Asturias and Instituto Universitario de Oncología del Principado de Asturias, Universidad de Oviedo, Oviedo, Spain; ^3^ Department of Molecular Oncology, Hospital Universitario Central de Asturias and Instituto Universitario de Oncología del Principado de Asturias, Universidad de Oviedo, Oviedo, Spain; ^4^ Department of Pathology, Hospital Universitario Central de Asturias and Instituto Universitario de Oncología del Principado de Asturias, Oviedo, Spain; ^5^ Biostatistics Unit, Hospital Universitario Central de Asturias, Oviedo, Spain; ^6^ Department of Pathology, Columbia University Medical Center, New York, NY, USA; ^7^ Departments of Otolaryngology/Head and Neck Surgery, Columbia University Medical Center, New York, NY, USA

**Keywords:** HNSCC, prognosis, PIK3CA mutation, S6 phosphorylation, immunohistochemistry

## Abstract

The PI3K/AKT/mTOR signaling pathway has emerged as one of the most frequently deregulated in head and neck squamous cell carcinomas (HNSCC). Numerous alterations of various upstream and downstream components have been described; however, their prognostic significance and impact on HNSCC patient survival remains to be established. This was addressed using an unbiased cohort of 93 consecutive and homogeneous surgically treated HNSCC patients and results confirmed in 432 HNSCC patients. Our findings reveal the high prevalence of S6 phosphorylation, a surrogate marker of mTORC1 activation, in HNSCC specimens (>70%) and, more importantly, demonstrate its relevance on clinical outcome. Phosphorylation of ribosomal protein S6 on either Ser235/236 or Ser240/244 was consistently and significantly correlated with favorable prognosis, although with differences depending on the tumor site. Thus, p-S6 expression was significantly correlated with better disease-specific survival specifically in the subgroup of laryngeal carcinoma patients (*P*< 0.001). In addition, multivariate regression models revealed p-S6 to be an inverse and independent predictor of lymph-node metastasis (*P*= 0.004) and distant metastasis (*P*= 0.006). Taken together, this study unveils an unprecedented correlation of mTOR activation with improved clinical outcome in patients with laryngeal carcinomas and uncovers the potential of p-S6 expression as a good prognostic biomarker and an inverse predictor of lymph node and distant metastases. These results should be of broad interest as immunohistochemical detection of p-S6 may help to stratify patients and guide treatment decisions.

## INTRODUCTION

Head and neck squamous cell carcinoma (HNSCC) is the sixth most common cancer worldwide. The term HNSCC is applied primarily to those cancers arising from the squamous epithelium of the oral cavity, oropharynx, hypopharynx, and larynx. Patients with HNSCC have benefited greatly from the latest advances in surgical techniques, radiation therapy and chemotherapy. However, despite advances in local control and overall quality-of-life achieved with the use of combined modality therapies, the survival rates for HNSCC have only marginally improved over the past two decades [1]. Hence, novel methods of cancer detection and prognosis need to be developed.

HNSCC is a heterogeneous disease involving deregulation of multiple pathways linked to cellular differentiation, cell cycle control, apoptosis, angiogenesis, and metastasis [2]. The PI3K/AKT/mTOR signaling pathway has emerged as one of the most frequently altered in multiple cancers including HNSCC [3–5] and multiple upstream and downstream components such as Epidermal Growth Factor Receptor (EGFR), phosphatidylinositol 3-kinase (PI3K), protein kinase B (AKT/PKB), phosphatase and tensin homolog (PTEN) and mammalian target of rapamycin (mTOR) have been found deregulated, making this pathway very attractive for the development of molecular-targeted therapies [6].

The PI3K pathway has been found genetically deregulated in human cancers at various levels. The first identified genetic mechanism of PI3K pathway activation was the loss of PTEN function by mutation or deletion, leading to the accumulation of the PI3K product phosphatidylinositol (3,4,5)-trisphosphate (PIP_3_). The accumulation of PIP_3_ activates a signaling cascade starting with the phosphorylation (activation) of the protein serine-threonine kinase AKT by 3-phosphoinositide dependent protein kinase-1 (PDK1/PDPK1). PDK1 has been considered a “master kinase” that phosphorylates and is responsible for the activation of other ACG kinase family members, many of them related to cell proliferation, survival or the inhibition of apoptosis, including AKT1, 2 and 3 [7].

Recent studies have reported high frequencies of somatic mutations in the PI3K catalytic subunit, p110α gene (*PIK3CA*), in various cancer types including HNSCC [8, 9]. The majority of *PIK3CA* mutations have been clustered within the helical (exon 9) and catalytic (exon 20) protein domains [8]. Furthermore, three hot-spot mutations have been identified: E542K, E545K (both mapping in exon 9) and H1047R (in exon 20), which have been shown to increase PI3K oncogenic activity and to confer transforming properties *in vitro* and *in vivo* [10]. The *PIK3CA* mutation rates reported in HNSCC studies range from 2.6% to 19% [5], found to be particularly common in HPV-positive oropharyngeal tumors (reaching 24-28%) [11].

Activating *AKT1* mutations have also been described in solid tumors [12, 13]. The E17K mutation in the Pleckstrin homology (PH) domain of the *AKT1* gene can result in PI3K-independent membrane recruitment of AKT, recapitulating the effects of the AKT8 murine leukemia retrovirus GAG-AKT fusion protein. E17K-*AKT1* exhibits transforming activity *in vitro* and *in vivo*, albeit at lower level than the myristoylated AKT [13]. A case of *AKT1* mutation has been recently documented in a patient with high-risk HNSCC [14].

Nevertheless, the prognostic significance of PI3K/AKT/mTOR pathway alterations and their impact on HNSCC patient survival remains to be established. This prompted us to perform a thorough analysis of PI3K/AKT/mTOR pathway components to investigate the mutational status of *PIK3CA* and *AKT1* genes and the immunohistochemical expression of a number of pathway-related proteins (EGFR, PDK1, p-AKT, PTEN, p-S6) using a consecutive series of 93 surgically treated HNSCC patients, and to establish correlations with the clinicopathological parameters and disease outcome. The PI3K/AKT/mTOR pathway contains numerous putative therapeutic targets; however, to determine the prevalence and clinical relevance of molecular alterations in this pathway is fundamental to identify those with potential prognostic and therapeutic application to HNSCC patients.

## RESULTS

### Significance of *PIK3CA* and *AKT1* mutations in HNSCC patients

The presence of the three activating hot-spot mutations (E542K, E545K and H1047R) in exons 9 and 20 of the *PIK3CA* gene and the oncogenic E17K mutation in the PH domain of the *AKT1* gene was analyzed in laryngeal and hypopharyngeal squamous cell carcinoma tissue specimens by mutant-enriched sequencing (Supplementary Figure S1). *PIK3CA* mutations were detected in 10 cases (13%); nine cases harboring only c.3140A>G (H1047R) mutation and one case harboring both H1047R and c.1624G>A (E542K). The *AKT1* c.49G>A (E17K) mutation was detected in seven cases (9%) and in two cases AKT1 mutation coexisted with *PIK3CA* (H1047R) mutation.

The relationships between *PIK3CA* c.3140A>G (H1047R) mutation, *AKT1* c.49G>A (E17K) mutation and the clinicopathological variables are shown in Supplementary Table S1. *PIK3CA* (H1047R) mutation was more frequent in laryngeal carcinomas (20%, 8 cases in larynx *vs* 6%, 2 cases in hypopharynx) and was significantly associated with stage I-II tumors (*P* = 0.002), and negative lymph node metastasis (*P* = 0.003).

In addition, *PIK3CA* (H1047R) mutation was more frequently detected in non-recurrent tumors (5 out of 6 cases; *P* = 0.077) and only 1 tumor with local recurrence, whereas tumors with regional recurrence or distant metastasis did not harbor *PIK3CA* (H1047R) mutation. Univariate Kaplan-Meier analysis showed that patients with *PIK3CA* (H1047R) mutation experienced a better disease-specific survival almost reaching statistical significance (Log-rank test, *P* = 0.063). Due to the complexity of our mutant-enriched sequencing methodology for routine clinical analysis, we also assessed the presence of *PIK3CA* mutations with two different kits used for diagnostic purposes in clinical laboratories (i.e. cobas® 4800 PIK3CA Mutation Test by Roche Molecular Diagnostics and PIK3CA Mutation Analysis Kit by EntroGen). While results obtained with both diagnostic kits perfectly matched, *PIK3CA* mutations were detected at a much lower frequency (1 positive case, 2%) than using the mutant-enriched sequencing methods (10 positive cases, 13%).

On the other hand, the presence of *AKT1* (E17K) mutation in this HNSCC series did not show influence on prognosis (Supplementary Table S1) or patient survival (Log-rank test, *P* = 0.427).

### S6 phosphorylation is a common feature in HNSCC patients that correlates significantly with improved clinical outcome in laryngeal carcinomas

**Figure 1:**
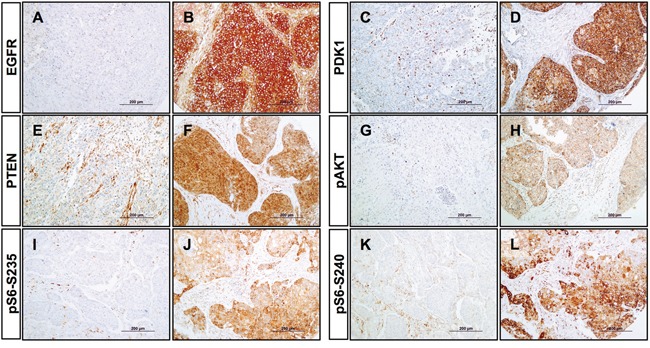
Immunohistochemical analysis of PI3K pathway proteins in HNSCC tissue specimens Representative examples of negative and positive expression of EGFR **A, B.** PDK1 **C, D.** PTEN **E, F.** phosphorylation of AKT on Ser473 **G, H.** phosphorylation of S6 on Ser235/236 **I, J.** and phosphorylation of S6 on Ser240/244 **K, L**. Original magnification x200.

The activation status of the PI3K/AKT/mTOR pathway was investigated in our series of 93 laryngeal and hypopharyngeal carcinomas by analyzing the immunohistochemical expression of multiple upstream and downstream components, such as EGFR, PDK1, PTEN, p-AKT and p-S6. Positive EGFR expression was found in 16% (12/75) cases (Figure 1A and 1B) and showed no correlation with clinical parameters. Positive PDK1 expression was detected in 26 (35%) out of 75 patients (Figure 1C and 1D). PDK1 expression did not correlate significantly with any of the clinicopathological features. Loss of PTEN expression was found in 40% (30/75) tumors (Figure 1E and 1F). PTEN loss tended to correlate inversely with lymph node involvement in this cohort (Fisher's Exact test; *P* = 0.081). Since PTEN is an inhibitor of the PI3K/AKT/mTOR pathway, this supports an association of PTEN loss with favorable prognosis.

We also analyzed the phosphorylation status of AKT at Ser473 and ribosomal protein S6 at both Ser235/236 and Ser240/244. Positive p-AKT expression was observed in 45% (34/75) cases (Figure 1G and 1H); however, no correlations with clinical data were found. Activation of ribosomal protein S6 through phosphorylation on either Serine 235/236 or Serine 240/244 was detected in 68% (51/75) and 77% (58/75) tumors, respectively (Figure 1I–L). More importantly, positive p-S6 expression (in particular Ser235/236) was significantly correlated with clinicopathological parameters associated with better prognosis (Table 1). Thus, p-S6 (Ser235/236) was significantly associated with negative lymph node metastasis (Fisher's Exact test; *P* < 0.001); smaller tumor size (*P* = 0.013); and lower disease stages (*P* = 0.001). Moreover, patients showing S6 phosphorylation on Serine 235/236 exhibited lower risk of tumor recurrence (*P* = 0.006), and significantly less distant metastasis (*P* = 0.003) and tumor-associated death (*P* = 0.002).

**Table 1:** Associations of p-S6 protein expression with clinicopathological findings, relapse and disease outcome

Characteristic	No.	p-S6(Ser235) expression (%)	*P*^†^	p-S6(Ser240) expression (%)	*P*^†^
**- pT classification**					
T1-T2	29	25 (86)	0.013	26 (90)	0.118
T3	25	16 (64)		18 (72)	
T4	21	10 (48)		14 (67)	
**- pN classification**					
N0	27	26 (96)	<0.001	25 (93)	0.022
N1-3	48	25 (52)		33 (69)	
**- Disease stage**					
I-II	15	15 (100)	0.001	15 (100)	0.023
III	14	12 (86)		12 (86)	
IV	46	24 (52)		31 (67)	
**- Pathological grade**					
Well differentiated	28	22 (79)	0.308	25 (89)	0.161
Moderately differentiated	33	20 (61)		23 (70)	
Poorly differentiated	14	9 (64)		10 (71)	
**- Site**					
Hypopharynx	34	20 (59)	0.142	23 (68)	0.097
Larynx	41	31 (76)		35 (85)	
**- Tumor Recurrence (at five years)**^‡^	25	21 (84)	0.006	21 (84)	0.085
No	32	15 (47)		20 (63)	
Yes					
**- Disease status (at five years)**					
Alive without disease	27	23 (85)	0.002	23 (85)	0.043
Dead of index cancer	30	13 (43)		18 (60)	
___					
Died of other causes	18	15 (83)		17 (94)	
**Total Cases**	75	51 (68)		58 (77)	

† Fisher's exact test.

‡ Patients who died from causes not related to the index tumor were excluded from the recurrence analysis.

Furthermore, a multivariate regression model was constructed in order to evaluate the influence of the following molecular and clinicopathological parameters on lymph node involvement in an independent fashion: tumor size, localization, pathological grade, PTEN expression, PDK1 expression, *PIK3CA* (H1047R) mutation and S6 phosphorylation. Hypopharyngeal localization, positive PTEN expression, absence of *PIK3CA* (H1047R) mutation, and negative p-S6 (Ser235/236) showed an independent predictive value for lymph node metastasis. According to these data, the risk for nodal metastasis was estimated to be 5.8-fold higher in patients with hypopharyngeal tumors (HR = 5.82; CI 95% 1.32-25.64; *P* = 0.02), 4.7-fold higher in patients with positive PTEN expression (HR = 4.7; CI 95% 1.17-18.78; *P* = 0.028), 13.65-fold higher in patients without *PIK3CA* (H1047R) mutation (HR = 13.65; CI 95% 1.36-137.38; *P* = 0.026), and remarkably 49.6-fold higher in patients with negative p-S6 (Ser235/236) tumors (HR = 49.61; CI 95% 3.43-716.11; *P* = 0.004).

Interestingly, using analogous multivariate models we found that S6 phosphorylation (Ser235/236) was the only significant independent predictor for distant metastasis. Thus, patients with negative p-S6 (Ser235/236) tumors exhibited 7.6-fold higher risk to develop distant metastasis than positive p-S6 (Ser235/236) tumors (HR = 7.66; CI 95% 1.81-32.47; *P* = 0.006).

**Figure 2:**
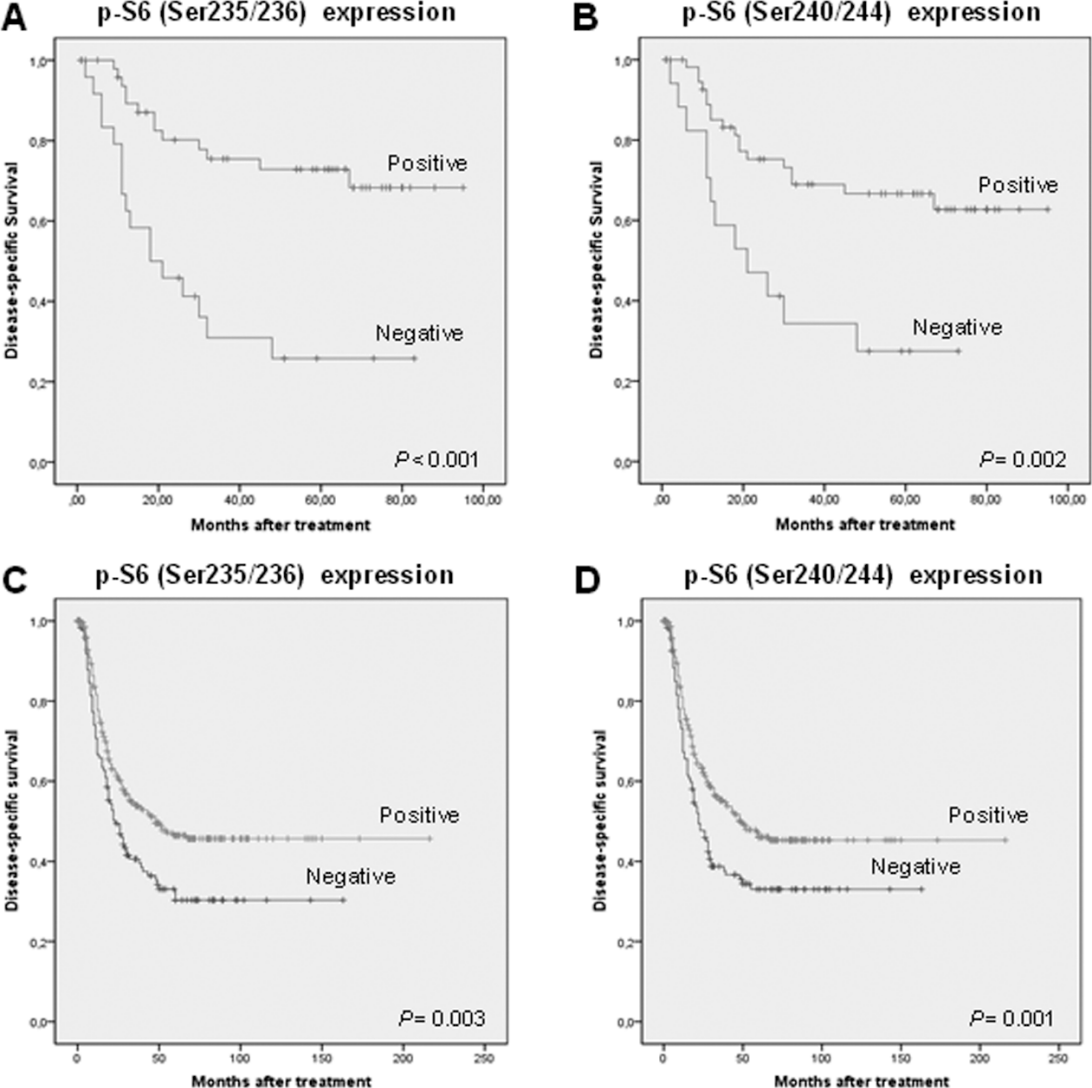
mTOR activation is associated with favorable prognosis in HNSCC patients Kaplan-Meier disease-specific survival curves of the 93 patients included in the study categorized by S6 phosphorylation on Ser235/236 **A.** and S6 phosphorylation on Ser240/244 **B.** Kaplan-Meier disease-specific survival for the extended series of 432 HNSCC patients categorized by S6 phosphorylation on S235/236 **C.** and S6 phosphorylation on Ser240/244 **D.** *P* values were estimated using the log-rank test.

We next examined the influence of PI3K pathway components on patients' survival. Univariate Kaplan-Meier analysis showed that patients with tumors harboring S6 phosphorylation in either Ser235/236 or Ser240/244 had a significantly better disease-specific survival (Log-rank test, *P* < 0.001 and *P* = 0.002 respectively, Figure 2A and 2B). In addition, the results obtained from univariate Cox analysis are also shown in Table 2. Furthermore, multivariate Cox analysis was performed including tumor size, localization, lymph node metastasis, pathological grade, and p-S6 (Ser235/236) (Table 3). This model showed that only a negative p-S6 (Ser235/236) expression was a significant independent predictor of reduced disease-specific survival (HR = 2.47; CI 95% 1.11-5.5; *P* = 0.026), and the presence of nodal metastasis was near significant (HR = 3.1; CI 95% 0.97-9.89; *P* = 0.056).

**Table 2:** Univariate Cox regression analysis for the disease-free survival (DSS) and the overall survival (OS)

Parameter	5-year DSS	HR (95%CI)	*P*	5-year OS	HR (95%CI)	*P*
**Localization**						
Larynx	70%	1		56%	1	
Hypopharynx	41%	2.19 (1.06-4.55)	0.035	38%	1.8 (0.96-3.36)	0.06
**T classification**						
T1-T2	68%	1		54%	1	
T3-T4	50%	1.63 (0.74-3.55)	0.21	44%	1.2 (0.63-2.26)	0.59
**N classification**						
N0	87%	1		69%	1	
N1-3	41%	4.89 (1.7-14.1)	0.003	35%	2.7 (1.26-5.64)	0.01
**Pathological grade**						
G1-G2	62%	1		52%	1	
G3	35%	2.02 (0.89-4.55)	0.11	31%	1.7 (0.69-3.5)	0.18
**p-S6 (Ser235/236)**						
Positive	73%	1		60%	1	
Negative	26%	3.93 (1.89-8.16)	<0.001	21%	2.6 (1.38-4.89)	0.004
**p-S6 (Ser240/244)**						
Positive	67%	1		54%	1	
Negative	26%	2.96 (1.41-6.19)	0.006	27%	2 (1.01-3.99)	0.045

HR Hazard Ratio, CI Confidence Interval

**Table 3:** Multivariate Cox regression analysis for the disease-free survival (DSS) and the overall survival (OS)

Parameter	HR (95% CI) DSS	*P*	HR (95%CI) OS	*P*
**Localization**				
Larynx	1		1	
Hypopharynx	1.19 (0.54-2.65)	0.66	1.16 (0.58-2.35)	0.67
**T classification**				
T1-T2	1		1	
T3-T4	1.07 (0.48-2.38)	0.88	1.17 (0.6-2.28)	0.64
**N classification**				
N0	1		1	
N1-3	3.01 (0.97-9.89)	0.056	1.99 (0.86-4.59)	0.11
**Pathological grade**				
G1-G2	1		1	
G3	1.61 (0.71-3.69)	0.25	1,42 (0.67-3.03)	0.36
**p-S6 (Ser235/236)**				
Positive	1		1	
Negative	2.47 (1.11-5.5)	0.026	2.6 (1.38-4.89)	0.003

HR Hazard Ratio, CI Confidence Interval

**Figure 3:**
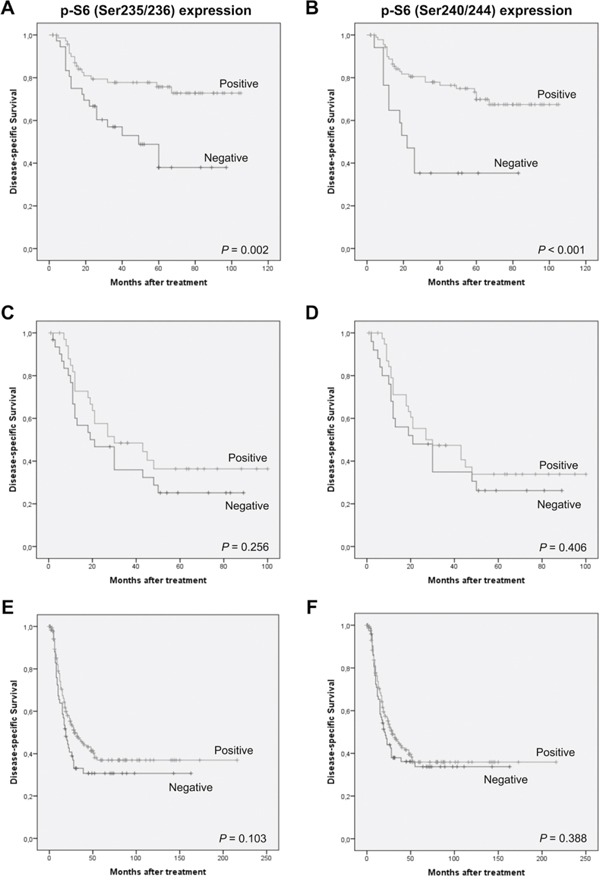
Distinctive effect of S6 phosphorylation on patients' survival depending on the tumor site Kaplan-Meier disease-specific survival curves categorized by S6 phosphorylation on Ser235/236 and S6 phosphorylation on Ser240/244 on the subgroup of laryngeal **A, B.** hypopharyngeal **C, D.** and oropharyngeal carcinomas **E, F**.

To further confirm these data, immunohistochemical analysis of both p-S6 (Ser235/236) and (Ser240/244) was extended to a larger series of HNSCC patients including 432 additional cases from various tumor sites (oropharynx, larynx and hypopharynx). Analogous results were obtained in this extended patient cohort, confirming the relationship of S6 phosphorylation with clinicopathological parameters associated with a better prognosis (Supplementary Table S2) and improved disease-specific survival (Log-rank test, *P* = 0.003 and *P* = 0.001, Figure 2C and 2D). The impact of p-S6 expression on disease course was also examined separately in each anatomic site, as we noted differences in p-S6 expression among the various tumor locations. Clear differences were observed on patient survival between the different subgroups. Specifically, we found that patients harboring laryngeal tumors with S6 phosphorylation on either Ser235/236 or Ser240/244 exhibited a significantly improved disease-specific survival (Log-rank test, *P* = 0.002 and *P* < 0.001, Figure 3A and 3B), whereas differences did not reach statistical significance in the subgroups of hypopharyngeal and oropharyngeal carcinomas (Figure 3C–3F). Nevertheless, p-S6 expression also tended to associate with a better survival in these subgroups of patients. The results from Univariate Cox analysis in the laryngeal subgroup also showed that both p-S6 (Ser235/236) and (Ser240/244) were strongly and significantly associated with improved disease-specific survival and overall survival (Table 4), although p-S6 expression was not found to be a significant independent predictor in multivariate analysis (HR = 1.24; CI 95% 0.65-2.32; *P* = 0.51 for Ser235/236, and HR = 1.04; CI 95% 0.45-2.38; *P* = 0.93 for Ser240/244).

**Table 4:** Univariate Cox regression analysis for the disease-free survival (DSS) and the overall survival (OS) in the laryngeal subgroup of patients

Parameter	5-year DSS	HR (95%CI)	*P*	5-year OS	HR (95%CI)	*P*
**T classification**						
T1-T2	88%	1		72%	1	
T3-T4	51%	4.47 (1.85-10.81)	0.21	38%	2.52 (1.35-4.59)	0.003
**N classification**						
N0	85%	1		72%	1	
N1-3	33%	6.53 (3.03-14.08)	<0.001	28%	4.18 (2.29-7.42)	<0.001
**Pathological grade**						
G1-G2	70%	1		59%	1	
G3	31%	3.47 (1.68-7.16)	0.001	19%	2.74 (1.44-5.2)	0.002
**p-S6 (Ser235/236)**						
Positive	76%	1		64%	1	
Negative	38%	2.67 (1.38-5.17)	0.003	28%	2.27 (1.31-3.93)	0.003
**p-S6 (Ser240/244)**						
Positive	70%	1		61%	1	
Negative	35%	3.34 (1.62-6.87)	0.001	25%	2.72 (1.43-5.16)	0.002

HR Hazard Ratio, CI Confidence Interval

Together these data confirm the prevalence of S6 phosphorylation and consequently mTORC1 activation in HNSCC tissue specimens and also its clinical relevance on patient prognosis and disease outcome, particularly in the subgroup of laryngeal carcinoma patients.

## DISCUSSION

Recent advances in deep sequencing have uncovered the complexity and heterogeneity of the HNSCC oncogenome [5, 15]. Despite the high diversity of genetic alterations underlying each individual tumor, most molecular alterations converge into few commonly deregulated pathways [16]. The PI3K/AKT/mTOR signaling pathway has emerged as the most frequently mutated mitogenic pathway in HNSCC (31%) with multiple actionable targets, representing an excellent opportunity to develop more personalized therapeutic strategies [5, 16]. Nevertheless, the identification of clinically and biologically relevant features in this pathway is fundamental to define the central nodes that may be exploited therapeutically. Hence, using an unbiased cohort of consecutively and homogeneously treated (surgery) HNSCC patients, we conducted a comprehensive study to investigate the prognostic significance of multiple genetic and biochemical alterations in key players of the PI3K/AKT/mTOR pathway (i.e. *PIK3CA* and *AKT1* mutations and immunohistochemical expression of EGFR, PDK1, p-AKT, PTEN and p-S6). Collectively, our results suggest a positive correlation between the mTORC1 activation and a less aggressive phenotype, resulting in a significantly improved survival in the subgroup of laryngeal carcinomas.

Remarkably, our findings reveal the high prevalence of S6 phosphorylation, a surrogate marker of mTORC1 activation, in HNSCC specimens (>70%) and, more importantly, demonstrate for the first time its relevance on clinical outcome: phosphorylation of ribosomal protein S6 on either Ser235/236 or Ser240/244 was a significant inverse predictor of lymph node metastasis, regional recurrence and distant metastasis. Consequently, S6 phosphorylation was also correlated with improved disease-specific survival in these patients, although the differences were only significant in the subgroup of laryngeal carcinomas. Hence, measurement of S6 phosphorylation by immunohistochemistry or by other means may help to stratify patients and/or to guide treatment decisions.

Activating *PIK3CA* (H1047R) mutation was the most frequently found mutation in our series (13%, 10 cases), particularly frequent in the larynx (20%, 8 cases). Moreover, *PIK3CA* (H1047R) mutation was associated with a better clinical outcome in laryngeal and hypopharyngeal carcinoma patients. Since most of these mutations (8/10) were present in laryngeal cancers, this association could be attributable to this location. In line with this, a recent analysis of TCGA sequencing data showed that *PIK3CA* mutations tended to associate with improved survival in HNSCC patients [17].

Numerous evidences have associated activating *PIK3CA* mutations with a more tumorigenic phenotype [18, 19]; however, contradicting results have been obtained when evaluating the prognostic significance of *PIK3CA* mutations in different human cancers. Thus, an association with favorable prognosis has been demonstrated in patients with breast [20] and esophageal carcinomas [21], whereas a poor prognosis has been reported in lung, colon and rectum cancer [19, 22, 23].

Nevertheless, *PIK3CA* (H1047R) mutation rate observed in our series by mutant-enriched sequencing could not be confirmed by *PIK3CA* mutation analysis with two different kits routinely used for diagnostic purposes. Thus, *PIK3CA* mutations were detected at a much lower frequency (1 positive case, 2%) than using the mutant-enriched sequencing methods (10 positive cases, 13%). These differences may be attributable to a higher sensitivity of the mutant-enriched method (approximately 0.25%), as we previously reported [24], questioning the prognostic relevance of these data, due to the low mutation rate observed at the sensitivity level (1-5%) of current routine diagnostic methods.

These results clearly indicate that despite activating mutations may be partially contributing, they are not a major driving force for the frequent PI3K/AKT/mTOR pathway activation detected in HNSCC. Although the PI3K pathway has been reported to harbor the highest percentage of mutations (30.5%) [5], widespread genomic and epigenetic alterations may account for the frequent activation of the PI3K/AKT/mTOR axis in HNSCC. Various plausible mechanisms have been thoroughly discussed by Iglesias-Bartolome *et al*. [16]. In addition to activating *PIK3CA* mutations, copy number gains and mRNA overexpression are also frequent events in HNSCC (at 20% and 52%, respectively). In a previous study, we detected *PIK3CA* gene amplification in 37% fresh HNSCC samples, and *AKT2* gene amplification in 30% tumors [3]. In addition, the present study also showed frequent PTEN loss and p-AKT in 40% and 45% cases respectively, which all may result in the activation of downstream targets such as mTOR. It is also worth noting that none of these upstream components individually or combined had an impact on patient survival in our series. Consequently, they are unlikely to be responsible for the good prognosis associated to the sustained mTORC1 activation, as reflected by S6 phosphorylation. Alternatively, it has also been demonstrated that there is a crosstalk between PI3K/AKT/mTOR pathway and the RAS/RAF/MEK/ERK pathway in cancers [25]. Furthermore, another interesting possibility of specific relevance to HNSCC is that REDD1, SESTRIN1 and SESTRIN2, all downstream targets of p53 and negative regulators of mTOR, are presumably disabled in more than 50% of HNSCC harboring *TP53* mutations what could result in mTOR activation in the absence of any other obvious genomic alteration of the PI3K pathway.

Various pathway activation phenotypes have been described in cancer and normal cells that could potentially explain the association with improved prognosis, including oncogene-induced senescence, maintenance of differentiation, and anti-invasive/anti-metastatic phenotypes [26–28]. Concordant with our *in vivo* data on HNSCC patients, these observations strongly and consistently reinforce that mTORC1 activation in the context of HNSCC may elicit anti-invasive and anti-metastatic phenotypes in a subset of patients.

mTOR activity has been associated to increased cell motility and metastasis in colorectal cancer, prostate cancer and lymphangioleiomyomatosis (LAM)[29, 30]. In addition, mTOR inhibition has been found to either decrease or promote cell migration and invasion depending on the cell system [29, 31, 32], thus indicating that mTOR activation only results in increased metastatic capability in a very specific cellular context. The fact that HNSCCs with mTORC1 activation (p-S6-positive) have a comparatively better survival than those with less active mTORC1, does not preclude the potential therapeutic value of PI3K/mTOR inhibitors for HNSCCs. In fact, extensive work has been performed defining the pathway may represent a viable molecular target in this tumor type. Rapamycin treatment has been used in different oncogenic HNSCCs models and seems to constitute a therapeutic alternative, for at least a subset of patients [16, 33–35]. In addition, multiple PI3K/AKT/mTOR inhibitors have been developed targeting different nodes of this pathway, mainly PI3K isoforms, AKT, mTORC1 and /or mTORC2 complexes, which are currently into clinical trials. In future work, it would be of interest to investigate if HNSCCs with p-S6-positive phenotype are more sensitive to PI3K/mTOR inhibitors than p-S6-negative tumors.

The clinical impact of hyperactive mTOR depends on the tumor type as it has been revealed in two recent meta-analyses examining data from numerous previous studies [36, 37]. The combined message of these meta-analyses is that hyperactive mTOR associates with worse prognosis in gynecological and gastrointestinal cancers; it is not associated with worse prognosis in breast cancer or even associated with good prognosis in some breast cancer types [38] or in lung cancer [36]; and it is inconclusive (due to small sample sizes) in HNSCCs and prostate cancers. Our results provide further evidence showing that mTOR activation (by means of p-S6 expression) has a distinct impact on patient survival depending on the tumor localization.

In relation to the subsite-specific effect of pS6, although we cannot provide a precise explanation on the underlying mechanism of this differential effect, remarkably, analogous observations have been reported for other molecular alterations in a recent analysis of HNSCC genomic alterations using TCGA data [17], and also in previous studies from our group [39, 40]. Thus, a number of molecular alterations (such as Cortactin, Anoctamin 1, *EGFR* amplification or even *PIK3CA* mutation) have been shown to have a differential impact on patient survival specifically in the larynx. Therefore, this underscores that, in addition to molecular alterations, local anatomical circumstances also play a role in patient outcome. Further investigation into the biologic differences between larynx and other subsites should be performed.

To summarize, our study unveils an unprecedented correlation between mTOR activation and favorable prognosis and improved clinical outcome in laryngeal carcinoma patients, and uncovers the potential of S6 phosphorylation as a good prognostic biomarker and an inverse predictor of lymph node and distant metastases. Activation of the PI3K/AKT/mTOR pathway is considered to play a crucial role in human neoplasms and so numerous drugs targeting this pathway have been developed and are currently in clinical trials [6, 41–43] including HNSCC (reviewed in [44]. Therefore, our results should be of broad interest, as immunohistochemical detection of p-S6 may help to stratify patients and guide treatment decisions.

## MATERIALS AND METHODS

### Patients and tissue specimens

Surgical tissue specimens from 93 patients with laryngeal and hypopharyngeal squamous cell carcinoma who underwent surgical treatment at the Hospital Universitario Central de Asturias were collected, in accordance to approved institutional review board guidelines. All experimental protocols were approved by the Institutional Ethics Committee of the Hospital Universitario Central de Asturias. Informed consent was obtained from all patients. Representative tissue sections were obtained from archival, formalin-fixed and paraffin-embedded (FFPE) blocks and the histological diagnosis was confirmed by an experienced pathologist (AA).

This series included all patients with laryngeal and hypopharyngeal carcinomas consecutively treated between 2002 and 2005, who met the following criteria: a single primary tumor, microscopically clear surgical margins, absence of distant metastasis at the time of diagnosis and no treatment prior to surgery. Only three patients were women, the mean age was 60 years (range 36 to 86 years). All but four patients (96%) were habitual tobacco and alcohol consumers. 41 (44%) patients received postoperative radiotherapy (this was administered to pT4 and/or pN2-N3 patients). The characteristics of the patients studied and the clinicopathological features of their tumors (site, pT classification, pN classification, disease stage, and pathological grade) are shown in Supplementary Table S1. The stage of disease was determined after the surgical resection of the tumor according to the TNM system of the International Union against Cancer (7th Edition). The histological grade was determined according to the degree of differentiation of the tumor (Broders' classification).

The median follow-up of the whole series was 33 months (range, 1-95 months), and the median follow-up of the patients alive at the last visit was 68 months (range, 51-95 months). Data on HPV status were available for the whole series, as previously described [45, 46]. As HPV infection may be a confounding factor, two HPV-positive patients were excluded from all the analyses.

In addition, a large cohort of 432 homogeneous surgically treated HNSCC patients was selected as a validation series. This series included patients with laryngeal, oropharyngeal and hypopharyngeal carcinomas treated at the Hospital Universitario Central de Asturias between 1990 and 2009, who met the same inclusion criteria above-described for the exploratory series. All patients had a single primary tumor, microscopically clear surgical margins and received no treatment prior to surgery. Only fourteen patients were women, and the mean age was 59 years (range 33 to 86 years). All but twelve (97%) patients were habitual tobacco smokers and 404 were alcohol drinkers. The characteristics of the patients studied and the clinicopathological features of their tumors are shown in Supplementary Table S2. 244 (56%) of 432 patients received postoperative radiotherapy. Only HPV-negative patients were included in the analysis.

### Tissue microarray construction

Five morphologically representative areas were selected from each individual paraffin-embedded tumor block: two for DNA isolation and three for the construction of a tissue microarray (TMA). To avoid cross-contamination, two punches of 2 mm diameter were taken first, using a new, sterile punch (Kai Europe GmbH, Solingen, Germany) for every tissue block, and stored in Eppendorf tubes at room temperature to be used for DNA extraction. Subsequently, three 1 mm tissue cylinders were taken from each of the 93 HNSCC to construct TMA blocks, as described previously [47]. In addition, each TMA also contained three cores of normal epithelium as an internal control. In order to check the histopathologic diagnosis and the adequacy of tissue sampling, a section from each TMA was stained with hematoxylin and eosin and examined by light microscopy. The same procedure was employed to construct TMA blocks from all the HNSCC patients included in the validation cohort.

### DNA extraction and mutational analysis of *PIK3CA* and *AKT1* genes

FFPE tumor cylinders were firstly deparaffinized in xylene and rehydrated through graded ethanol solutions. Subsequently, the tissue pellets were digested in lysis buffer containing 2 μg/μL proteinase K and the genomic DNA isolated using the QIAamp DNA Mini Kit (Qiagen, Valencia, CA) following manufacturer's instructions.

Mutations in exons 9 and 20 of the *PIK3CA* gene and the E17K hot-spot mutation of the *AKT1* gene were analyzed by direct genomic sequencing methods and confirmed by using mutant-enriched sequencing methods that we previously developed [48]. All PCR fragments were purified using ExoSAP-IT kit (Affymetrix, Santa Clara, CA) and sequencing was performed with ABI Prism 3730xl DNA analyzers by Genewiz, Inc (South Plainfield, NJ). Any alteration detected was further verified by sequencing of a second PCR product derived independently from the original DNA template.

*PIK3CA* mutations were also assessed using two different kits designed for diagnostic purposes i.e. cobas® 4800 PIK3CA Mutation Test by Roche Molecular Diagnostics (Branchburg, NJ) and PIK3CA Mutation Analysis Kit by EntroGen (Los Angeles, CA), following manufacturer's protocols.

### Immunohistochemistry

The HNSCC TMAs were cut into 3-μm sections and dried on Flex IHC microscope slides (Dako). The sections were deparaffinized with standard xylene and hydrated through graded alcohols into water. Antigen retrieval was performed using Envision Flex Target Retrieval solution, high pH (Dako) or with proteinase K (for EGFR). Staining was done at room temperature on an automatic staining workstation (Dako Autostainer Plus) using the Dako EnVision Flex + Visualization System (Dako Autostainer) and the following antibodies: EGFR pharmDx (TM) kit (Dako), mouse monoclonal anti-PKB kinase (PDK1) (E-3; Santa Cruz; sc-17765) at 1:2000 dilution, rabbit monoclonal anti-Human phospho-Akt (Ser473), Phosphorylation Site Specific, (Clone 14-5; Dako; M3628) at 1:20 dilution, rabbit anti-phospho-S6 Ribosomal Protein (Ser235/236) (Cell Signaling # 2211) at 1:200 dilution, rabbit monoclonal Phospho-S6 Ribosomal Protein (Ser240/244) (D68F8 XP®; Cell Signaling # 5364) at 1:200 dilution, and mouse monoclonal anti-Human PTEN (Clone 6H2.1; Dako; M3627) at 1:50 dilution. Counterstaining with hematoxylin was the final step.

The slides were viewed randomly, without clinical data, by two of the authors. The average intra- and inter-observer variation was <5%. Since EGFR, PDK1 and PTEN staining showed a homogeneous distribution, a semi-quantitative scoring system based on staining intensity was applied. Thus, EGFR immunostaining was scored as negative (0), weak (1), moderate (2) and strong protein expression (3) and PDK1 and PTEN were scored as negative (0), weak (1), strong (2) staining. For statistical purposes, EGFR and PDK1 staining data were dichotomized as negative expression (scores 0-1) *versus* positive expression (scores >1) and for PTEN score 0 was considered loss of PTEN expression.

Immunostaining for p-AKT and p-S6 was evaluated using a semi-quantitative scale (0%, <10%, 10-50%, or >50% positive tumor cells). For statistical analysis, staining data were dichotomized as negative expression (0-10% stained cells) *versus* positive expression (>10% stained cells), previously established as p-AKT cut-off level [49].

### Statistical analyses

All the statistical analyses were performed using complete data sets. The experimental results distributed among the different clinical groups of tumors were tested for significance employing the Fisher's exact test. Logistic regression models were constructed to evaluate the independent effect (Odds Ratio) of the molecular variables on a given clinicopathological feature. Survival curves were calculated using the Kaplan-Meier product limit estimate. Differences between survival times were analyzed by the log-rank method and the Hazard Ratio (with 95% CI) was calculated by univariate Cox regression analysis. Multivariate Cox proportional hazard models were used in a stepwise manner, and each variable was tested with the other factors as a co-variable. The HR with 95% CI and *P* values were reported. All tests were two-sided. The values of *P* ≤ 0.05 were considered statistically significant.

## SUPPLEMENTARY FIGURES AND TABLES


