# Supplementary material for: Impact of PI3K/AKT/mTOR pathway activation on the prognosis of patients with head and neck squamous cell carcinomas
Source: Oncotarget. 2016 Apr 23;7(20):29780–93. doi: 10.18632/oncotarget.8957 (PMC5045433; doi:10.18632/oncotarget.8957)
Supplement: Supplementary file 1 [file oncotarget-07-29780-s001.pdf]

Impact of PI3K/AKT/mTOR pathway activation on the prognosis of patients with head and neck squamous cell carcinomas

SUPPLEMENTARY FIGURE AND TABLES

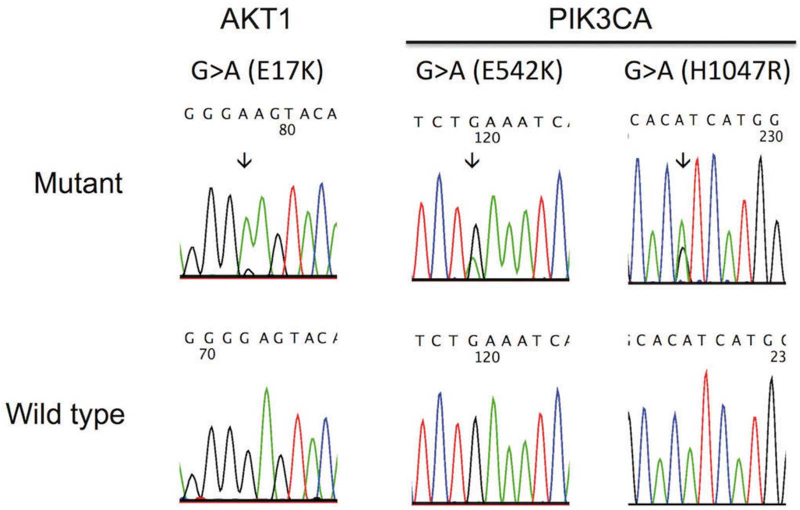

Supplementary Figure S1: Sequencing results showing *AKT1* (E17K) and *PIK3CA* (E542K and H1047R) hot-spot mutations found in HNSCC patients.

Supplementary Table S1: Associations of *PIK3CA* (H1047R) and *AKT1* (E17K) mutations with clinicopathological findings, relapse and disease outcome

Characteristic	No.	*PIK3CA* (H1047R) Mutation (%)	*P*†	*AKT1* (E17K) Mutation (%)	*P*†
**- pT classification**					
T1-T2	29	6 (21)	0.327	2 (7)	0.019
T3	25	2 (8)		0 (0)	
T4	21	2 (9)		5 (24)	
**- pN classification**					
N0	27	8 (30)	0.003	4 (15)	0.244
N1-3	48	2 (4)		3 (6)	
**- Disease stage**					
I-II	15	6 (40)	0.002	2 (13)	0.396
III	14	2 (14)		0 (0)	
IV	46	2 (4)		5 (11)	
**- Pathological grade**					
Well differentiated	28	7 (25)	0.072	4 (14)	0.324
Moderately differentiated	33	2 (6)		3 (9)	
Poorly differentiated	14	1 (7)		0 (0)	
**- Site**					
Hypopharynx	34	2 (6)	0.101	4 (12)	0.695
Larynx	41	8 (20)		3 (7)	
**- Tumor Recurrence (at five years)‡**					
No	25	5 (20)	0.077	1 (4)	1.000
Yes	32	1 (3)		2 (6)	
**- Disease status (at five years)**					
Alive without disease	27	5 (19)	0.091	1 (4)	1.000
Dead of index cancer	30	1 (3)		2 (7)	
___					
Died of other causes	18	4 (22)		4 (22)	
**Total Cases**	75	10 (13)		7 (9)	

† Fisher's exact test.

‡ Patients who died from causes not related to the index tumor were excluded from the recurrence analysis.

Supplementary Table S2: Associations of p-S6 protein expression with clinicopathological findings, relapse and disease outcome in the validation series of HNSCC patients

Characteristic	No.	p-S6(Ser235) expression (%)	*P*†	p-S6(Ser240) expression (%)	*P*†
**- pT classification**					
T1-T2	137	110 (80)	0.003	115 (84)	0.001
T3	143	93 (65)		95 (66)	
T4	139	88 (63)		91 (65)	
**- pN classification**					
N0	133	104 (78)	0.007	107 (80)	0.005
N1-3	289	188 (65)		194 (67)	
**- Disease stage**					
I-II	65	60 (92)	<0.001	62 (96)	<0.001
III	74	55 (74)		56 (76)	
IV	283	177 (63)		183 (65)	
**- Pathological grade**					
Well differentiated	178	139 (78)	0.003	136 (76)	0.136
Moderately differentiated	161	102 (63)		109 (68)	
Poorly differentiated	82	50 (61)		55 (67)	
**- Site**					
Oropharynx	245	183 (75)	0.004	167 (68)	0.004
Hypopharynx	68	37 (54)		43 (63)	
Larynx	109	72 (66)		91 (83)	
**- Tumor Recurrence‡**					
No	104	80 (77)	0.016	83 (80)	0.009
Yes	221	140 (63)		144 (65)	
**- Disease status**					
Alive without disease	113	88 (78)	0.004	90 (80)	0.005
Dead of index cancer	212	132 (62)		137 (65)	
___ Died of other causes	97	69 (71)		74 (76)	
**Total Cases**	422	292 (69)		301 (71)	

Only HPV-negative patients were included in the analysis.
† Fisher's exact test.
‡ Patients who died from causes not related to the index tumor were excluded from the recurrence analysis.
